# Using time use diaries to assess physical activity and sedentary behavior in jail populations

**DOI:** 10.1186/s12889-025-23706-z

**Published:** 2025-07-18

**Authors:** Ricky Camplain, Sara Shuman, Adrienne Alonso, Elizabeth Schmitter, Javier Lopez, Amy Gelatt, Rebecca Annorbah, Isabel Fangman, Morgan Occhino, Brooke de Heer, Dirk de Heer, Meredith Brown, Kate Compton-Gore, Travis Pinn, Beya Thayer, Richard Martin, Linnea Evans

**Affiliations:** 1 Department of Epidemiology and Biostatistics, School of Public Health, Indiana University, 1025 E 7th St, Bloomington, Bloomington, IN 47405 USA; 2 Center for Community Health and Engaged Research, Northern Arizona University, Flagstaff, AZ USA; 3 Southwest Institute for Research on Women, University of Arizona, Tucson, AZ USA; 4 Department of Health Sciences, Northern Arizona University, Flagstaff, AZ USA; 5 Department of Criminology and Criminal Justice, Northern Arizona University, Flagstaff, AZ USA; 6 Yavapai County Sheriff’s Office, Camp Verde, AZ USA; 7 Yavapai Justice and Mental Health Coalition, Yavapai County, St. Presscott, AZ USA; 8 Department of Health Promotion and Policy, University of Massachusetts– Amherst, Amherst, MA USA

**Keywords:** Incarceration, Physical activity measurement, Sedentary time measurement, Time use patterns, Daily diaries, Movement behaviors

## Abstract

**Background:**

People incarcerated have limited access to physical activity and there are challenges to accurately measure their physical activity in jail settings. We aimed to (1) determine the feasibility of time use diaries to measure physical activity and sedentary time among people incarcerated in jail and (2) estimate time spent in physical activity and sedentary behaviors, overall and by gender and job assignment.

**Methods:**

In July 2023, we recruited women and men from two housing units at Yavapai County Detention Facility, a county jail in Camp Verde, Arizona. Participants were asked to complete a four-day time use diary, documenting activities in 10-minute increments. Participants provided feedback about the diary during listening sessions after the four-day period. Feasibility was measured as the proportion of people recruited who participated and the proportion of completed and returned diaries. Activities documented in the diaries were linked to the Compendium of Physical Activities to determine intensity and duration of physical activity and sedentary behavior. Average daily time spent in physical activity and sedentary behavior was calculated for each participant.

**Results:**

Of the 24 women and 41 men recruited, 24 (100%) women and 40 (97.5%) men agreed to participate in the study. All diaries were returned. Most (82.8%) participants completed all four days in the diary. Participants were enthusiastic about filling out the diaries because they helped pass the time and felt the work would further knowledge and wellbeing in jails. Participants made concrete recommendations including altering the diary format to allow for additional detail about their time in jail. Participants spent on average 571 minutes sedentary, 79 minutes in light activity, and 60 minutes in moderate activity per day. Women spent 66 more minutes per day engaged in light activities compared to men. Women with job assignments spent 179 less minutes in sedentary behavior and 245 more minutes in moderate activity compared to women who did not have a job assignment.

**Conclusions:**

Time use diaries are a feasible and appropriate way to measure physical activity and sedentary behavior among people incarcerated in jail.

## Background

At any given time in the United States, over 750,000 people are incarcerated in jails, short-term detention facilities that hold people in pre-trial detention and convicted of misdemeanor level charges [1, 2]. Although considered “short-term,” individuals can be incarcerated for months or even years in jails. Those who are convicted can serve sentences up to one year and those who have not been convicted and are awaiting trial may be held in jail for months or even years.

Many individuals who experience incarceration are also at greater risk of having chronic health conditions, substance use disorders, and poor mental health prior to being incarcerated, because of societal stratification processes. Carceral environments can exacerbate pre-existing health conditions as well as accelerate the onset of new health concerns among individuals with no prior health concerns [3, 4]. In some cases, the consequences can be short-term (e.g., momentary feelings of high-stress and sleep loss), whereas others may be more persistent and lead to long-term health concerns (e.g., prolonged anxiety/depression, weight gain). People entering jail may experience excess stress, anxiety, and depression [5, 6], which may lead to poor sleep and increases in blood pressure [7]. A single bout of moderate-to-vigorous physical activity can reduce anxiety and depression symptoms [8, 9], decrease blood pressure [8, 10], and improve sleep quality [8, 11] on the day it is performed. Disease risk reduction and improved physical functioning accrue within days after adopting a new physical activity routine [8, 12], primarily through regulation of body temperature, adrenal activity, and neurotransmission of noradrenaline and dopamine [13, 14]. Body regulations can contribute to short-term calming effects, stress adaptation, and improved mood. Although the advantages of physical activity are clear, people incarcerated in jail have limited opportunities to participate in physical activity to experience the benefits [15, 16]. 

Jails may provide leisure-time opportunities to be physically active; however, these activities are voluntary and usually unstructured. Individuals incarcerated may have access to recreation time, an unstructured time dedicated to physical activity, often outside. Programs available to individuals incarcerated (e.g., substance abuse treatment) may incorporate structured physical activity opportunities such as yoga or other exercise classes. However, depending on a jail’s policies and practices, they may not provide these opportunities or individuals may choose not to participate. Previous research has found that when recreation time is provided, 75% of individuals incarcerated reported they do not attend [17]. Of women who attend recreation time, 58% were sedentary during recreation time [18]. 

Another way individuals report accumulating physical activity while incarcerated is during work-related activities. Job assignments are common in US jails with over half of individuals incarcerated assigned to some type of work during incarceration [19, 20]. However, it is important to note that not everyone qualifies for a job assignment while incarcerated. The type of charge, as well as behavioral assessments often dictate job assignment eligibility. Importantly, in one study that did investigate perceptions on physical activity, women who worked while incarcerated reported sufficient physical activity to meet the 2018 Physical Activity Guidelines for Americans [8, 21]. 

Although studies have shown that people incarcerated in jails and prisons are sedentary and engage in limited physical activity, there is little physical activity and sedentary behavior research among people incarcerated in jail [22]. Limited research is due, in part, to the difficulty of measuring movement behaviors while incarcerated. Accelerometers, the gold standard of physical activity and sedentary behavior measurement, have traditional disadvantages such as lack of context of participants’ activities. Accelerometers also have correctional setting-specific disadvantages as the technology is considered contraband in many facilities and therefore cannot be used. Self-reported questionnaires, widely used to estimate physical activity and sedentary behavior in the general populations, are low cost and easily distributed [23, 24]. However, of the numerous questionnaires available to measure physical activity [25], many are context-specific, limiting the potential for use in the jail population. Even the International Physical Activity Questionnaire, developed to easily compare physical activity across different populations, is not designed for correctional settings [26]. Further, time estimate questions where respondents are asked to estimate the amount of time they spend in a specific activity often result in overestimates of time spent in physical activity. Direct observation, such as the System for Observing Play and Recreation in Communities (SOPARC), is another way to measure movement behaviors through an objective method of collecting information in a specific environment. SOPARC was adapted to adequately measure the level of activity of people incarcerated during specific programs, such as recreation time [27]. However, systematic observation is not feasible on a large scale or for daily activity estimates.

Time use diaries are a data collection method through which people indicate uninterrupted, sequential accounts of continuous streams of activities, together with estimated start and finish times, throughout a specified observation period. Time use diaries accurately estimate time-use patterns and are appropriate for population-level estimates of physical activity and sedentary behavior, specifically time spent in leisure and household physical activity and sedentary behavior [28, 29]. However, time use diaries have not been assessed for use in correctional facilities. Thus, we aimed to employ a novel approach to measuring movement behaviors in a correctional setting by determining the feasibility and acceptability of time use diaries to measure physical activity and sedentary time among people incarcerated in jail. We additionally aimed to conduct a preliminary assessment of physical activity and sedentary behavior among individuals incarcerated in the jail, overall as well as by sex and employment status.

## Methods

### Study design, setting, and sample

To determine if time use diaries are a feasible way to measure physical activity and sedentary behavior among people incarcerated in jail, we conducted a cross-sectional study. In July 2023, we recruited participants from Yavapai County Detention Center (YCDC), a jail in Camp Verde, Arizona. Yavapai County is a rural county that is home to three Native American reservations, a high percentage of White and Latino(a) populations, and persons living in poverty. At the time of the study, YCDC housed an average of 535 sentenced and un-sentenced individuals in six pods with 22 housing units. Individuals are confined to their assigned housing unit, which are segmented by sex assigned at intake, risk assessment evaluation, and known conflicts. If an individual is required or allowed to be somewhere outside of their housing unit, they must be escorted by a detention officer or other jail staff member.

The jail primarily has unstructured days, with few organized activities for incarcerated individuals. Meals are provided on a structured schedule: breakfast at 4:30 AM, lunch at 11:00 AM, and dinner at 4:00 PM. Individuals incarcerated have opportunities to engage in volunteer-led programs, including religious studies, high school equivalency courses, and behavioral health initiatives. Recreation time is offered “as often as possible”. Individuals incarcerated are also allowed to use tablets, through which they may access self-help courses, guided coursework, literature, books, movies and TV shows, music, and games. However, accessing these resources on the tablets incurs a cost for the individual. Specific to physical activity, individuals can access exercise videos and other physical activity programs.

Individuals are eligible to work in the jail if they meet certain criteria: (1) they are convicted of their charges and sentenced to time in the jail, (2) they meet behavior requirements (e.g., no infractions while incarcerated), and (3) they are not convicted of a violent crime. Men who meet these criteria can work in the kitchen or perform maintenance tasks around the facility, such as lawn work. Men with assigned work duties are housed in a separate unit from non-workers. Because women constitute only about 15-20% of the jail population and must be separated from men, those who meet the criteria can be assigned to a laundry job assignment. Women with assigned work duties are housed with non-workers.

We recruited participants using a convenience sampling strategy. YCDC leadership independently chose one male and one female general population housing unit for recruitment. All people incarcerated in the selected housing units were eligible to participate in the study. All participants provided informed consent, and all study activities were approved by Northern Arizona University’s Institutional Review Board (#2171602), which included a “Prisoner Advocate.”

### Recruitment procedures

Three study team members were escorted into the women’s housing unit by a YCDC staff member and a detention officer. In the housing unit’s day room, the research team introduced the study. If a woman was not interested in participating, they were asked to return to their individual cell. The research team reviewed the consent materials in a group setting, allowing for questions through the presentation. At the end, women in the unit were given another chance to return to their cell if they declined to participate in the study. If they agreed to participate in the study, women individually signed and returned the consent form signature page and completed a short pencil-and-paper demographic questionnaire. The study team talked participants through the instructions on how to fill out the four-day time use diary, explained the process for participating in listening sessions at the end of the four-day period, and answered questions. Following completion of recruitment in the women’s housing unit, the study team repeated the same recruitment process in the men’s housing unit.

### Demographic questionnaire

Participants were asked when they were admitted into the jail, their age, ethnicity, and race. We asked participants if they had a job assignment at the time of recruitment which job they were assigned for the purpose of accurately recording physical activity related to job duties.

### Time use diaries

**Fig. 1:**
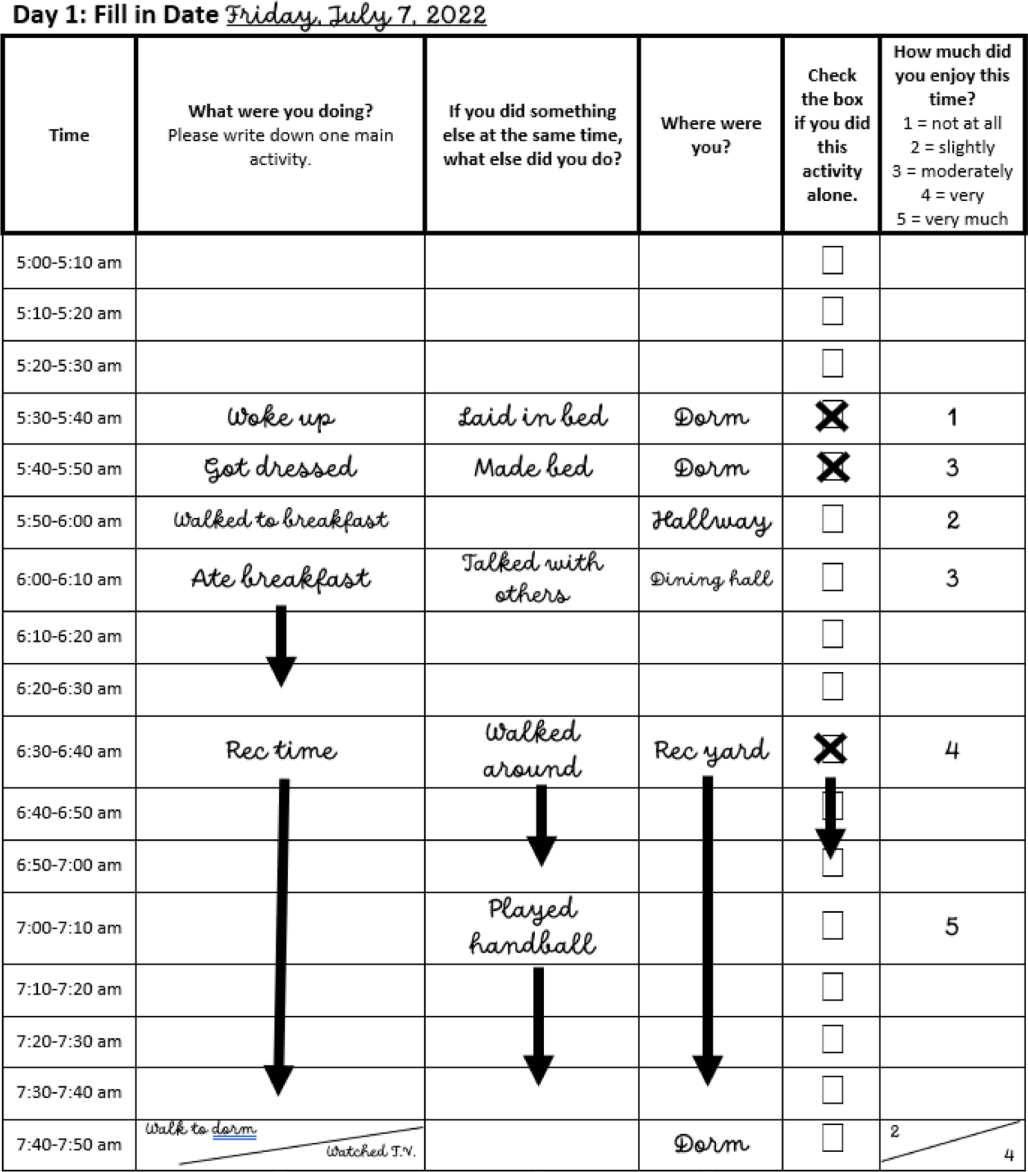
Time use diary example

Time use diaries are activity logs in which a participant is given a four-day diary and instructed to record their activities in “their own words” starting from 4 a.m.−4 a.m. the next day, in 10-minute increments. The sequential and comprehensive accounting of daily activities is considered the most reliable and accurate accounting of daily-time, as it aligns with the way daily events are stored in memory and requires respondents to arrange time within the constraint of a 24-hour period [30, 31]. Over the course of four days, the participants completed a pencil-and-paper time use diary (Fig. 1) using golf pencils provided by the facility. Participants reported what they were doing in 10-minute increments. Participants described the main activity (primary activity) and simultaneous activity (secondary activity) they engaged in during the indicated 10-minute increment. They additionally reported their location in the jail during the activity, if they did the activity alone, and rated how much they enjoyed the activity on a 5-point scale. A reflection page was included at the end of the diary with the prompt: “What thoughts or feelings did you experience today when completing the diary? Is there anything else you feel is important to share about your day?” Diaries were bound with glue and met the jail’s materials requirements.

### Listening sessions

After four days, the research team returned to the housing units to collect the completed time use diaries and to conduct listening sessions with participants. During the listening sessions, all participants from each dorm gathered in the dayroom. The facilitator asked open ended questions about their experience with the diaries. Questions included questions about how participants approached filling out the diary, what they liked and disliked about filling out the diary, challenges they had while filling out the diary, recommended changes to the diaries, and how they felt while filling out the diaries. Listening sessions were not recorded. Two researchers took detailed notes in pre-printed notebooks with dedicated pages for each question. Notetakers had separate sections of notes for each listening session. Notes were combined and researchers who were present during listening sessions reviewed notes for accuracy and met as a group to discuss changes to the notes page. Notes were summarized into main topics.

### Compensation

Each participant received pre-packaged commissary items worth $45 when the study was completed. If a participant was released before the end of the study, they received an e-gift card worth $45. If a participant was transferred to the Department of Corrections before the end of the study, we sent $45 to their prison commissary account.

### Measuring physical activity and sedentary behavior

The Compendium of Physical Activities is a list of physical activities and sedentary behaviors used to code the type, purpose, and intensity of activities (metabolic equivalents or METs) in everyday life [32, 33]. Three researchers independently assigned each primary and secondary activity to a Compendium activity that most closely related to the activity in the diaries. Previous research has shown that objectivity in using the Compendium to code activities is high (objectivity coefficients > 0.80) and remained adequate with just one coder [34]. Accordingly, all coders coded separate diary entries. Following individual coding, one coder reviewed all codes, documented all changes made, and discussed all changes with the group. Any coding conflicts were resolved by consensus. All ambiguous and unknown activities were documented by researchers, discussed as a group, and resolved by consensus twice during the coding process. Location of an activity, if indicated by a participant, was often used to decipher an activity. Once Compendium activity codes were finalized, the time use diary data was linked to the Compendium using the original Compendium crosswalk file. Consequently, each non-sleep 10-minute interval was assigned a MET value and physical activity level: light activity (1.6-3 METs), moderate activity (3–6 METs), vigorous activity (> 6 METs), or sedentary activity (1-1.6 METs). Diary days were excluded from analyses if there were any time intervals without an activity in the 4:00 AM – 4:00 AM day. Average daily and weekly time spent in light, moderate, and vigorous physical activity and sedentary behavior was estimated for each participant. Further, we determined if participants met the 2018 aerobic Physical Activity Guidelines for Americans [8]. We estimated the number of minutes a participant spent in moderate and vigorous physical activity over seven days by taking the average time spent in moderate and vigorous physical activity and multiplying by seven days. If an estimate was 150 min of moderate physical activity, 75 min of vigorous physical activity, or an equivalent combination, then a participant met guidelines.

### Statistical analysis

We determined the proportion of women and men recruited who consented to be a participant in the study, proportion of returned diaries, and number of completed days.

Demographic information, including age, identity (race and ethnicity), whether a participant had a job assignment at the jail (e.g., laundry work), and how long participants had been incarcerated were assessed using descriptive statistics, including means, standard deviations (SD), frequencies and relative frequencies.

Differences in time spent in physical activity and sedentary behavior between men and women as well as by job assignment status (yes vs. no) among women were assessed using t-tests. Differences in meeting the 2018 Physical Activity Guidelines for Americans by sex assigned at intake and job assignment were determined using Chi-Square tests. An alpha level of 0.05 was used to determine statistical significance.

## Results

Of the 24 women and 41 men recruited, 24 women (100%) and 40 men (97.6%) agreed to participate in the study (Table 1). All (100%) diaries were returned, even if a participant was released or transferred before the end of the four-day study period. Most (52, 81.3%) participants completed all four days in the diary. Six (9.4%) participants completed three days, three (4.7%) completed two days, two (3.1.%) participant completed one day, and one (1.6%) participant did not have a complete diary for any of the four days. Thus, of the 260 possible days, participants completed 238 days (91.5%). A higher proportion of women completed all four days (92.0%) compared to men (76.9%). A higher proportion of non-working women completed all four days (94.7%) compared to working women (83.3%).

**Table 1:** Feasibility measures of time use diary use among women and men incarcerated in Yavapai County detention center (*n* = 64), July 2023

Feasibility Measure	All Participants *n* = 64		Men *n* = 39		Women *n* = 25					
					All Women *n* = 25		Non-Working Women *n* = 19		Working Women *n* = 6	
	*N*	%	*N*	%	*N*	%	*N*	%	*N*	%
Recruited ^a^										
Yes	64	98.5	39	97.5	25	100		100		100
No	1	1.5	1	2.5	0		0		0	
Returned Diaries										
Yes	64	100	39	100	25	100		100		10
No	0		0		0		0		0	
Number of completed diary days										
0	1	1.6	1	2.6	0		0		0	
1	2	3.1	2	5.1	0		0		0	
2	3	4.7	3	7.7	0		0		0	
3	6	9.4	4	10.3	2	8.0	1	5.3	1	16.7
4	52	81.3	29	74.4	23	92.0	18	94.7	5	83.3

^a^ 65 individuals (25 women and 40 men) were invited to participate in the study

### Sample characteristics

Over 90% of women were between 25 and 54 years old (Table 2). Only 64% of men were between 25 and 54 years with 15% of men in the youngest age group (18–24 years) and 21% in the oldest age group (≥ 55 years). In non-mutually exclusive categories, 78% of participants identified as White, 23% Hispanic/Latino(a), 20% Native American, and 5% Black. Identity was distributed similarly by sex assigned at intake. Women and men were incarcerated an average of 72 and 133 days, respectively (range: 1–701 days). Six (24%) women had a laundry job assignment while incarcerated. No men with a job assignment participated in our pilot study, a result of dorm selection.

**Table 2:** Demographic characteristics of women and men incarcerated in Yavapai County detention center (*n* = 64), July 2023

Characteristic	All Participants *n* = 64		Men *n* = 39		Women					
					All Women *n* = 25		Non-Working Women *n* = 19		Working Women *n* = 6	
	*N*	%	*N*	%	*N*	%	*N*	%	*N*	%
Age (years)										
Mean (SD)	38.9	13.4	39.8	15.2	37.6	10.2	37.6	10.5	37.7	10.1
18–24	7	10.9	6	15.4	1	4.0	1	5.3	0	
25–34	22	34.4	11	28.2	11	44.0	8	43.1	3	50.0
35–44	15	23.4	9	23.1	6	24.0	5	26.3	1	16.7
45–54	11	17.2	5	12.8	6	24.0	4	21.1	2	33.3
≥ 55	9	14.1	8	20.5	1	4.0	1	5.3	0	
Race and Ethnicity ^a^										
American Indian/Alaska Native	13	20.3	8	20.5	5	20.0	3	15.8	2	33.3
Black	3	4.7	2	5.13	1	4.0	1	5.3	0	
Hispanic/Latino(a)	15	23.4	10	25.6	5	20.0	4	21.1	1	16.7
White	50	78.1	30	76.9	20	80.0	16	84.2	4	66.7
Length of Incarceration										
Mean (SD)	108.8	(138.9)	133.1	(164.1)	71.9	(77.1)	61.6	66.5	104.3	104.4
Range (days)	1-701		1-701		11–289		11–289		22–246	
Q1 (1–22 days)	17	26.6	10	25.6	7	28.0	5	26.3	2	33.3
Q2 (23–44 days)	17	25.6	8	20.5	9	36.0	8	42.1	1	16.7
Q3 (45–167 days)	15	23.4	9	23.1	6	24.0	5	26.3	1	16.7
Q4 (168–701 days)	15	23.4	12	30.8	3	12.0	1	5.3	2	33.3

*Abbreviations*: *SD* Standard deviation

^a^ Not mutually exclusive

### Participant feedback (Listening sessions summary and reflection Pages)

During the listening sessions, men and women reported they approached filling out the diaries in two different ways: (1) every 10 min or (2) sitting down and recalling their previous activities. Participants sometimes found it difficult to complete the time use diary when using the second approach, as it was challenging to recall their past time use in 10-minute blocks if they were not able to fill out the diary consistently throughout the day. Other challenges included a limited ability to check the actual time, as participants are not permitted to have watches while incarcerated in the facility. Participants had to rely on either a stationary clock in the dorm’s day room that is not visible from their cells, or they had to check the time on tablets are that are checked-out during the day but must be returned at night.

“*… you will likely see people have the same activity listed at different times because we don’t always know what time it is*” – Female study participant.

When probed about what they liked and did not like about the time use diaries, participants said that filling out activities in 10-minute intervals was burdensome.

“*Did not like that it was every 10 minutes*” – Male study participant.

“*We can’t do it every 10 minutes. It slowed down time too much*” – Female study participant.

While participants acknowledged drawbacks to completing the diaries, they generally perceived the benefits to outweigh the disadvantages. Several participants indicated that the diaries provided a “*sense of purpose*” and made their days pass more quickly. However, it is important to acknowledge, in some cases, not all participants believed the research could result in systematic change.

“*This is like a little diary/agenda I like it! I was lost and lonely before you came here*,* I love this research I hope whatever it does for you guys helps your study*.” – Female study participant.

“*This was fun to do*.” – Female study participant.

“*While I thank you very much for the reward and the distraction I hope you don’t think you can make any difference to the bureaucratic inertia of this system*.” – Male participant.

During the listening session with the male participants, discussions emerged regarding the potential impact of their contributions. They expressed hope to contributing valuable knowledge that could enhance the health and well-being of future incarcerated individuals. Reflections from the diaries themselves also reflect contributions.

“*I liked this study I hope by doing this we can help improve the way things are ran for those incarcerated even in the slightest way. Thank you for allowing us to be part of your study*.” – Male study participant.

“*I want to thank you for giving us a voice to the outside world. Thank you.*” – Female study participant.

Overall, participants expressed enthusiasm about filling out the diaries and provided recommendations for improvement including longer intervals for recording their activities, additional reflection pages, more space in the daily log for the ability to provide more details in their diaries, and to provide a Spanish conversion for Spanish-speaking individuals.

“*More reflection space*” – Female study participant.

“*Add a spot for reflection every day*,* not just at the end*” – Male study participant.

### Preliminary Assessment - Physical activity and sedentary behavior

**Fig. 2:**
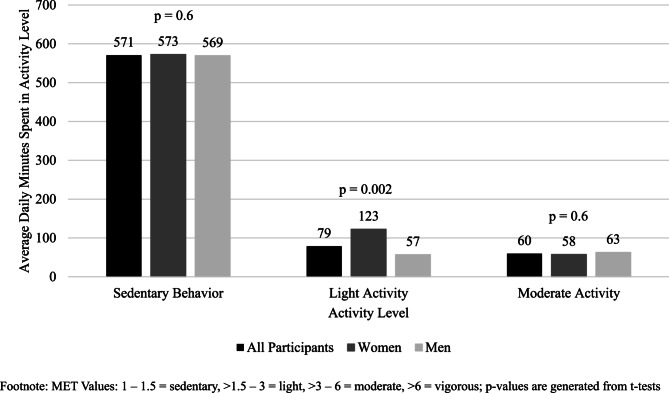
Average daily time (minutes) spent in activity level among women and men non-workers incarcerated in Yavapai county detention center (*n* = 58), July 2023

On average per day, non-worker participants spent 601 min (SD = 185) sedentary, 78 min (SD = 64) engaged in light activity, and 61 min (SD = 78) engaged in moderate activity per day (Fig. 2). There was only one 10-minute increment of vigorous physical activity among all diaries. Women (123 min, SD = 76) spent 67 more minutes per day in light activity compared to men (56 min, SD = 44.2, *p* = 0.002). Women reported more cleaning and grooming activities compared to men, which contributed to the difference in light activity. Although not statistically significant, women (586 min, SD = 141) spent 21 less minutes per day engaged in sedentary behavior compared to men (608 min, SD = 205, *p* = 0.6). Also not statistically significant, women (53 min, SD = 73, *p* = 0.6) spent 8 less minutes per day engaged in moderate activity compared to men (65, SD = 81, *p* = 0.6).

**Fig. 3:**
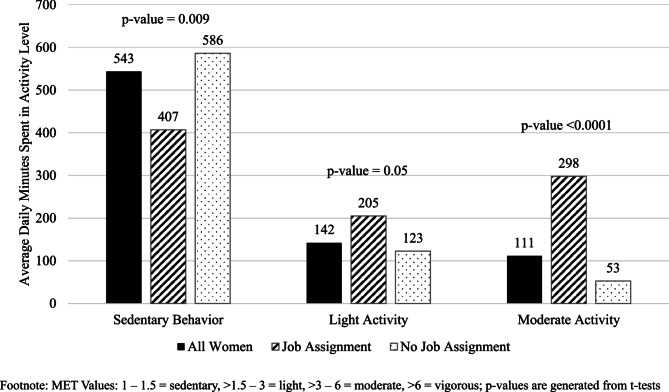
Average daily time (Minutes) spent in activity level by work status among women incarcerated in Yavapai County detention center (*n* = 25), July 2023

Because no men with a job assignment participated in the study, women were only included in work-related comparisons. Women, including workers and non-workers, on average per day, spent 543 min (SD = 153) in sedentary behavior, 142 min (SD = 91) in light activity, and 111 min (SD = 128) in moderate activity (Fig. 3). Most moderate activity among workers were job-related. Women workers (407 min, SD = 107) spent 179 less minutes per day in sedentary behavior compared to women non-workers (586 min, SD = 141, *p* = 0.009). Women workers (298 min, SD = 62) spent 227 more minutes in moderate activity compared to women non-workers (53 min, SD = 73, *p* < 0.0001). Although not statistically significant, women workers (205 min, SD = 114) spent more time in light activity compared to women non-workers (123 min, SD = 73, *p* = 0.05).

Among all participants, 61% met the 2018 Physical Activity Guidelines for Americans. There was no statistically significant difference in the proportion of participants who met the guidelines by sex assigned at intake. However, when assessing differences by job assignment among women, all six women with a job assignment met the guidelines (100%) compared to 47% of women without a job assignment (*p* = 0.02, data not shown).

## Discussion

Our study is the first to use time use diaries as a data collection instrument among people incarcerated. We assessed the feasibility and general acceptability of a pencil-and-paper self-completed time use diary among people incarcerated in jail. All but one person recruited agreed to participate in the study, all participants returned a time use diary to researchers, and over 80% of participants completed a diary for all four days. Participants were overall interested in the research topic and filling out the diaries. They were thoughtful with feedback and provided recommendations for improvement. We additionally explored preliminary patterns of physical activity and sedentary behavior among women and men incarcerated in jail. Participants spent most of their waking hours in sedentary behavior with less time dedicated to light and moderate activity. Only one participant engaged in any vigorous activity. The biggest differences in activity were between women laundry workers and women non-workers in which workers spent significantly more time in moderate activity and less time in sedentary behavior.

Previous research indicates time use diary data are less susceptible to reporting errors and social desirability bias than other physical activity questionnaires that ask people to estimate average time spent in an activity domain [28]. The American Time Use Survey Activity Lexicon activities have been previously linked to the compendium [35], and further validated using accelerometers and cameras [28]. Time use diaries estimate time in exercise and sedentary behaviors accurately when directly compared with camera records, individually and on a population level (e.g., mean) [28]. 

Although time use diaries are appropriate for measuring time in exercise, it is important to note job-related activities have been previously excluded from validity analyses as intensity categories of these activities could not be assigned to them using their coding scheme [28]. Being limited to measuring intensity of non-job-related activities limits the ability to capture overall physical activity and sedentary behavior among any population. However, because job-related physical activity has never been assessed among people incarcerated and a job assignment is often one of few opportunities for an individual incarcerated to engage in structured activities, it is important to accurately measure. Although our sample size was small, we were able to estimate MET values of job-related activities among 6 (11%) women. Women with job assignments had statistically significantly more light and moderate activity and less sedentary behavior compared to women without job assignments. Thus, it is imperative to accurately capture job-related activities when conducting physical activity and sedentary behavior research in jail. Due to our use of a convenience sampling strategy, where jail leadership selected one women’s dorm and one men’s dorm for participant recruitment, we were unable to include men with work duties in our sample, limiting our ability to make a fair overall comparison. Future research should include a sufficient sample size of men and women incarcerated with job assignments.

It is important to note that time use diaries are less dynamic than accelerometers as each activity is coded in 10-minute increments and has one MET value assigned to the entirety of an activity. Activities that participants engaged in for less than 10 min were rarely recorded. That may be especially important for more physically demanding job-related tasks, such as the laundry work. For example, if a laundry worker coded their diary for 3 h “at work”, the entire three hours were coded as “laundry worker” from the Compendium of Physical Activities with a MET value of 3.3 (moderate activity). Consequently, we may overestimate how much time individuals spend in activities. Similarly, walking to and from other locations in the jail, such as court, a work duty, or a visit do not require a 10-minute commute and overestimate time spent in walking activity. This may introduce differential misclassification of time as most participants did not indicate in their diaries that they walked from one location to another, even if the activity reported was clearly in a different location.

The potential overestimation of time spent in activities, particularly physical activities, may inflate the proportion of individuals who meet the 2018 aerobic Physical Activity Guidelines for Americans. Over 60% of participants and 100% of women with a job assignment met the aerobic guidelines, which is higher than the National Health Interview Survey estimate of about 47% of adults in the United States who met the aerobic guidelines [36]. Alternatively, most individuals incarcerated may be accumulating adequate amounts of physical activity to meet aerobic guidelines and potentially the health benefits that come with meeting guidelines. But it is important to note that people incarcerated experience worse health outcomes compared to their noninstitutionalized counterparts [5, 6]. Thus, the physical activity may not be enough to minimize the impact of stress and other negative consequences associated with incarceration. Both men and women incarcerated self-reported over 10 h of sedentary behavior, much higher compared to the 6–8 h among non-institutionalized Americans [37]. Sedentary behavior is an independent risk factor for poor health [38, 39], and strategies to improve health among people incarcerated may need to include reductions in sedentary behavior as well as increases in physical activity. Our current study only included a convenience sample of 64 adults incarcerated in one jail and did not include men with job assignments, individuals with severe mental illnesses, or individuals in administrative segregation. Those in administrative segregation do not have access to programs, recreation time, or a day room, where most individuals accumulated moderate physical activity. Our future research will determine at how physical activity and sedentary behavior may mitigate poor health outcomes, including stress, sleep, and blood pressure among a larger, more diverse sample of people incarcerated.

One significant challenge participants faced while filling out the time use diaries was staying engaged in reporting activities every 10 min, which was likely exacerbated by not always having access to a clock. Participants indicated it may be easier to complete diaries with longer time intervals. With challenges of accurately measuring not only physical activity and sedentary behavior, but also sleep, engagement in cognitively stimulating activities, and group activities, the research team found that lengthening time intervals would exacerbate the misclassification of activity types. Because participants were enthusiastic about the research, all participants were able to return a diary, and over 80% of participants completed all four days of the diary, we recommend that future research using time use diary methodology retain 10-minute intervals but emphasize the use of arrows in indicating activities that happen for longer than 10-minutes. Similarly, we will encourage participants to use slashes (/) to indicate that two primary activities occurred in the same 10-minute interval. Further, future research should ensure access to clocks as possible. Participants had access to the time on their tablets as well as on a wall clock and the phone kiosk in their day room. Therefore, it is our belief that misclassification in time was most likely to be during lockdown hours at night.

## Conclusions

Correctional facilities, including jails, are unique environments and require unique ways to measure physical activity and sedentary behaviors. Time use diaries are an appropriate and feasible way to collect physical activity and sedentary behavior among people incarcerated in jail. Other tools, such as non-specific physical activity questionnaires and accelerometers are not practical in the unique environment of correctional facilities. Future research should consider the use of time use diaries to estimate population-level physical activity and sedentary behavior patterns among individuals incarcerated. Our future research includes larger, more generalizable estimates of physical activity and sedentary behavior among people incarcerated in jail, with particular attention to differences in activities by work status. Further, we will assess how physical activity and sedentary behavior impact health outcomes while incarcerated.

## Data Availability

The data that support the findings of this study involve individuals who are currently or formerly incarcerated, a population considered protected under human subjects research regulations. Due to ethical and legal considerations, including the need to protect participant confidentiality and comply with institutional and regulatory requirements, the dataset is not publicly available. However, de-identified data may be made available upon reasonable request to the corresponding author, subject to approval by the appropriate institutional review board and data use agreements.
